# Interprofessional team training via telemedicine in medical and nursing education

**DOI:** 10.1186/s12909-024-06104-8

**Published:** 2024-10-08

**Authors:** Maria Härgestam, Hanna Morian, Lenita Lindgren

**Affiliations:** Department of Nursing, Umeå University, Umeå, Sweden

**Keywords:** Focus groups, Information communication technology, Medical students, Nursing students, Systems Engineering Initiative for Patient Safety, Interprofessional team training, Telemedicine

## Abstract

**Background:**

The use of information communication technologies such as telemedicine has increased over the years, offering access to specialized healthcare even in remote locations. However, telemedicine in interprofessional team training is seldom included in medical or nursing programs, and little is known about how to practise these scenarios. This study aimed to explore how medical and nursing students experience teamwork when one team member is participating remotely and digitally.

**Methods:**

Following interprofessional team training in which one team member participated remotely, focus group interviews were conducted with three teams, each comprising one medical student and two nursing students (*n* = 9 students in total). The focus group interviews were analysed with thematic content analysis. The Systems Engineering Initiative for Patient Safety model was applied as a theoretical framework and served as a lens in the analysis.

**Results:**

Three themes were identified in the analysis: *challenging the dynamic of leadership*, *becoming familiar with a new setting*, and *finding new strategies to communicate.*

**Conclusions:**

The results of this study suggest that future physicians and nurses need to enhance their knowledge of practicing teamwork through telemedicine during their education, as the use of telemedicine continues to grow.

**Supplementary Information:**

The online version contains supplementary material available at 10.1186/s12909-024-06104-8.

## Background

Providing equal healthcare is a key goal of Swedish healthcare [1]. However, implementing equal healthcare can be challenging, particularly in rural areas where access to specialized care can be limited and specialized physicians may not be readily available. For many years, information communication technology solutions such as telemedicine have served as valuable tools for consulting colleagues in different fields regarding patient care. In the late 1990s, a pilot study conducted in Sweden demonstrated a high level of satisfaction among both patients and physicians when utilizing telemedicine in rural areas [2]. The last 20 years of technological advancement have led to a more diverse use; hospitals can, for example, have X-rays analysed by physicians living in countries across different time zones [3]. For patients seeking primary care advice, new private internet-based telehealth commercial ventures are offering access to advice from physicians through different web-based applications accessible on mobile devices [4]. The growing availability and ease of use of these technological solutions have produced a high level of patient satisfaction and quality of care [5]. These new applications show promise in certain fields, with overall positive therapeutic effects, but further research is still needed [6, 7].

In emergency care, situations range from non-acute consultations to decisions requiring swift action, with an increased risk of the patient’s vital functions becoming unstable. Teamwork is a key aspect in caring for critically ill patients [8] and is emphasized in both medical and nursing education through interprofessional team training [9, 10]. This training is practised through theoretical and practical team training exercises at clinical training centres. However, these team training exercises are exclusively based on all team members being co-located.

As information communication technologies such as telemedicine are implemented in healthcare, it becomes important for students in nursing and medical education to practise collaboration and communication through these technologies. The Systems Engineering Initiative for Patient Safety model (SEIPS) is a theoretical framework that can be used in healthcare to enhance patient safety and improve overall system performance [11]. This model integrates work systems (including people, environments, tools, and tasks), work processes, and work outcome. The core idea of SEIPS is that these components interact dynamically in a way that can impact both patient and staff well-being. The model is particularly valuable for identifying how changes in one component can affect others, and for guiding the redesign of work systems to improve safety and efficiency. This framework can help clarify how healthcare personnel adapt to new technologies, such as teamwork through telemedicine. However, studies documenting teamwork via telemedical team training are scarce, and the main focus has been on the outcome and cost-effectiveness [12, 13]. Training is essential for working effectively via telemedicine [14, 15]. Currently, proficiency in telemedicine is a skill that must be developed on-site during students’ future professional practice. Despite limited hands-on experience among physicians and nurses, previous studies have demonstrated the usefulness of telemedicine in emergency care, showing a reduction in trauma transfers without compromising patient safety [12, 13]. To better understand simulation-based team training through telemedicine, the SEIPS framework can be valuable, as it integrates people (students and standardized patients), environments (care units and/or clinical training centres), tools (digital equipment), and tasks (simulation-based team training). Given the growing importance of telemedicine, further research is needed to develop and establish guidelines for implementing simulation-based team training in educational and workplace settings.

The aim of this study was to explore how medical and nursing students experience teamwork via telemedicine during simulation-based team training when one team member is participating remotely and digitally.

## Methods

This study is part of a research project conducted as a collaboration between Umeå University, Karolinska Institute, and Region Västerbotten.

### Setting and participants

Nine students, comprising three medical students and six nursing students from a university in Northern Sweden, were invited to participate in the study during their final year. The participants were recruited via email after approval from their respective heads of department. In the email, students were invited to contact the research group for more information. Inclusion criteria were being a registered student in a medical or nursing program and being over 18 years of age.

The recruited participants were aged between 24 and 27; six were female and three were male. All nine participants had experience of interprofessional team training during their education, and four had experiences of working in a distributed team i.e. geographically dispersed teams with members who collaborate using information and communication technology [16].

### Simulation-based team training reflected upon by participants in this study

The simulation-based team training is described in detail in Morian et al. [17]. In brief, each team participated in two 20-minute scripted scenarios involving a patient (an individual acting as a patient) with deteriorating vital signs at the Clinical Training Centre’s emergency room. The first scenario involved a patient with urosepsis, and the second was a myocardial infarction. Both scenarios occurred on the same day. In both scenarios, a standardized patient [18], trained to follow a script, was used to ensure consistency and encourage interaction. The scenarios were designed to escalate in severity at a predetermined time. When the patient’s condition worsened, the nursing students called the medical student at a predetermined trigger. During the scenarios, the nursing students were in the same room as the patient while the medical students participated remotely and digitally (i.e. via telemedicine), sitting in a room nearby. Zoom™, a video conferencing software—a synchronous audiovisual communication platform- was used to interact with the nursing and medical students. The screen was mounted on a mobile cart to allow easy movement and enable the remote doctor to view the patient from different angles.

The team training took place at the university’s clinical training centre and the focus group interviews were conducted directly after the team training.

### Data collection

Focus group interviews were conducted with three teams, each consisting of three participants: one medical student and two nursing students. The focus group interviews were carried out immediately after the team training by two of the authors (MH and HM), during autumn 2021 at the clinical training centre at Umeå University in Northern Sweden. The interviews were semi-structured, and began with a question inviting the participants to describe their experience of teamwork when one of the members was participating via telemedicine. HM led all the focus group interviews, and MH asked complementary questions when needed, made sure that all participants had the opportunity to speak up, and ensured that the time was distributed between the interviewees. A semi-structured interview guide was used (see Supplementary file), with open-ended questions such as “How did you perceive leadership in the distributed setting?”, “How did you perceive the collaboration in the distributed setting?”, and “Can you tell me how you experienced communication in the distributed setting?”, as well as follow-up questions such as “Could you please elaborate on this?” to help the participants deepen and expand their narratives. Focus group interviews make it possible to achieve an in-depth discussion about a particular area which takes place within the group [19]. Further follow-up questions were asked by the researchers when needed for clarification. The interviews were audio-recorded and lasted approximately 38 min each.

### Data analysis

The text was control-read and listened through initially to reduce the risk of misinterpretation during analysis. Following this, the transcribed focus group interviews were analysed using thematic analysis [20] which is a type of analysis that uses a step-by-step structured formula to increase replicability without losing flexibility. The analysis was conducted with an abductive approach, combining induction and deduction moving between the empirical material and theory [21]. The transcribed focus group interviews were read through repeatedly and individually by the authors in order to understand smaller segments and the interviews as a whole. The authors independently identified codes that corresponded to the purpose, and then the identified codes were compared and checked against the transcribed focus group interviews. Reoccurring codes were distilled into subthemes which were further distilled into themes and then compared to the full text to ensure they were representative. The authors continuously discussed the themes and sub-themes throughout the analysis process.

Finally, to gain an understanding of how the participants interacted with the technology, the themes were analysed in relation to SEIPS. This theoretical framework was applied as a lens in the analysis to explore how SEIPS can be understood in medical education from the perspective of nursing and medical students using information communication technologies. Considering the aim of this study, we chose to employ the concepts of people (the participants), task (team training), environment (the training centre), and tool (the screen) in the analysis. According to SEIPS, the interaction of these four concepts (people, task, environment, and tool) can affect work processes and work outcomes; this is highlighted in the results presented below.

## Results

The analysis was based on how the participants described the work process and work outcomes. Three major themes were identified: *challenging the dynamic of leadership*, *becoming familiar with a new setting*, and *finding new strategies to communicate*. In each theme, two subthemes were found in the analysis (Table 1). The interactions between people, environments, tools, and tasks were also analysed and related to work processes and work outcomes according to a simplified version of SEIPS known as SEIPS 101 [11]. Excerpts are included in the presentation of the results in order to contextualize and illustrate the participants’ descriptions, labelled by team number (FG) and course of study (NS = nursing student, MS = medical student).

**Table 1:** Overview of the themes and subthemes

Themes	Subthemes
Challenging the dynamic of leadership	Taking a step back to get an overview Ambiguous leadership role
Becoming familiar with a new setting	Facing new tasks and roles Transparency of knowledge is reassuring for the team
Finding new strategies to communicate	Compensating for the limited nonverbal communication Technological tools expand the environment

### Challenging the dynamic of leadership

Collaboration via a screen was described by the interviewees as creating new challenges but also advantages. The medical students who participated via the screen talked of “taking a step back” as a leader to ensure they had an overview of the situation. In the excerpt below, a medical student compared the situation to cardiac and pulmonary resuscitation (CPR):

*If you compare it to CPR*,* where you normally have one [person] with a good overview*,* another doing compressions*,* another giving medication*,* and one keeping an eye on the airways… And then one just standing there making sure everyone’s doing what they should. So it felt like I was in that situation. That I was like “Okay*,* have we done this? Good*,* then let’s do this now.” (FG1*,* MS1)*.

In this excerpt, the medical student described having an overview of the situation and time to plan for the next step of the patient’s care. The participants agreed that the leadership was not as clearly defined in these teams compared to their earlier experiences of teamwork in a co-located setting. Choosing the medical student as leader was not an active choice, but more an unspoken agreement. The medical student was perceived as medically responsible, and therefore also responsible for assigning tasks and carrying the teamwork forward. One of the nursing students concluded:

*I found it a good structure. You have the overview and it’s… it gets kind of natural for you to be the leader*,* to provide structure*,* and so… And then we like*,* share among us who does what. (FG1*,* NS1)*

As they were outside the emergency room (ER), the medical students felt less stressed than the nursing students who were situated with the patient in the ER. The medical students expressed that when the patients’ vital signs deteriorated in the first scenario, they could stay focused on their tasks; this was in contrast to the nursing students, who had to face the agitated patient and experienced more stress.

*Yeah*,* but that stress didn’t affect me in the same way. Instead I could check my notes and be like*,* okay*,* we’re at A*,* then we do this first. So that was nice. If I was there I wouldn’t have had the same freedom to have all these things around me that way. (FG1*,* MS1)*

Being present via the screen was perceived by the medical student as being by the patient’s side and still having access to the patient’s journal. This allowed the medical students to look up allergies and medical history without leaving the team.

Two groups pointed out that while the medical students were the overall leaders, the nursing students developed a dynamic of their own through swapping assignments between each other to better fit their strengths or current tasks. The nursing students found this aided workflow. One nursing student described it as follows:

*I feel a bit like I said before*,* that maybe it was more you taking the lead as physician. But it wasn’t fully that either*,* that you delegated to us like “Do this*,* do that.”… It was more like “You need to do this*,*” and then we divided it among us*,* like what came naturally. (FG2*,* NS2)*

The nursing students said that they had sometimes found the physician hard to reach during their previous experiences outside the study. Compared to this, the team training with telemedicine felt like an improvement because the medical student was available throughout the full scenario. This meant prescriptions and assignments could be changed on the go if, for instance, the patient needed more pain medication than was initially prescribed.

*Well*,* I felt that it was really reassuring and good*,* that you could have more contact with the physician for a long time*,* and there was always support*,* and you could double-check. (FG3*,* NS1)*

In terms of SEIPS, the team members (the people) described positive interactions via telemedicine (the tool), as the medical student was able to get an overview of the situation in the emergency room. This, in turn, could change the roles within the socio-organizational environment, affecting the workload differently. This had an impact on both the work process and the work outcome, as the medical students mentioned that participating via telemedicine could facilitate and contribute to a reduced workload. The nursing students described both barriers and facilitators; they experienced an enhanced workload due to having to step in as secondary leaders, while at the same time, they felt more secure with the medical student continuously present on the screen.

### Becoming familiar with a new setting

When working in a team with a remote team member, the nursing students encountered situations they did not recognize from previous experience of interprofessional teamwork. Some of their tasks were related to the nursing profession, but they also became partly responsible for examinations usually conducted by physicians.

*I think I could examine someone’s abdomen. We’ve practised that a little*,* I think I could do that. Some other things you’d need to practise… Like listening to the lungs maybe isn’t something you’ve really done a lot. But in that case it’s something you could practise. (FG1*,* NS1)*

In the excerpt above, the nursing student said that performing tasks commonly done by a physician was challenging since they lacked the “reference bank” that comes with having a lot of practise. The interviewees agreed that in order to ensure that the tasks were completed correctly and in a safe and secure manner, it was important for them to be open about their own knowledge and experience, and to ask for guidance and advice when performing unfamiliar tasks. Nevertheless, the medical students expressed that even though the nursing students had performed the assigned tasks correctly, they would have preferred to do them on their own.

*Well*,* it’s a bit like*,* when following ABCDE I would have preferred to listen to the lungs and heart on my own. Examined how the skin felt*,* pulse*,* and all that. I mean*,* it’s really not to do with not trusting what you say*,* but I think that*,* like*,* by doing*,* not just hearing*,* but instead seeing and feeling*,* you can get a better idea of the situation*,* so I think I would’ve done that. (FG2*,* MS1)*

In the excerpt above, the medical student pointed out the difficulties of trusting the nursing student’s examination of the patient. On the other hand, the nursing students described how they had to prioritize their tasks and change how they worked, since they “had fewer hands”. They expressed that at times they felt crowded with tasks, and lost their overview of the situation when they had to do examinations usually done by the physician. However, both medical and nursing students expressed that knowing the patient’s symptoms and suspecting a diagnosis could help with executing tasks and workflow, rather than relying on the medical student’s responsibility:

*… She arrives really anxious and nauseous and pressing her hands on her chest. I felt that ECG would definitely be needed here at some point*,* so I felt like I could be a step ahead in this and comfortable with that. (FG2*,* NS1)*

In terms of SEIPS, when working via telemedicine the nursing students (the people) described a change in their responsibilities and the tasks they were expected to perform. Their duties extended beyond tasks typically performed by the nursing profession, to also include physicians’ examinations. In this way, telemedicine (the tool) could be interpreted as a barrier to executing tasks more accurately. These factors influenced the work process. For nursing students, working remotely, i.e., when the patient and provider are not in the exact physical location and digitally, i.e., these remote services are delivered via video conferencing, meant being open about their own knowledge and experience and asking for guidance and advice when performing unfamiliar tasks. On the other hand, for the medical students it meant the added difficulty of trusting the nursing student’s examination of the patient, potentially affecting the work outcome.

### Finding new strategies to communicate

The interviewees concluded that the communication within the team needed to change when collaborating via telemedicine. One focus group described how they first tried to mimic a “normal” situation as much as possible and create opportunities for face-to-face communication. However, face-to-face communication with the medical student via the screen meant that the nursing students had to turn their backs on the patient, leaving them unattended. One of the nursing students narrated this as follows:

*I remember standing there feeling nervous*,* now she [the patient] was unattended behind us*,* and both of us were doing other things. (FG1*,* NS1)*

For the medical students, participating via telemedicine meant working with limited vision. They perceived this as a constraint, as it made it hard to have an overview and to know what the other team members were doing when they were out of sight. The interviewees mentioned that name badges were usually helpful, but recognized that in this situation, the medical student could only see the backs of the nursing students when they were interacting with the patient. This was deemed challenging, particularly within newly formed teams, when attempting direct communication and task assignment.

Another focus group expressed that it felt natural to work with direct communication using names and closed loops during the second scenario. One medical student said they were confident that vision and hearing were enough to communicate:

*I mean*,* if you can hear me then it’s like*,* that’s enough for us to communicate and that I see what you see. (FG3*,* MS1)*

Conversely, another medical student experienced that having the patient in sight provided an increased sense of control over the situation:

*The thing is*,* during the first case*,* I could like only see her [the patient’s] legs and that they were shaking*,* and I heard some whimpering and things like that. And that didn’t feel very good… I got a good report*,* and to start with*,* we were like “Okay*,* we need to do these things.”… And then you ran off*,* and I was left behind far away like “Hello? Is everything alright?” I felt like I had no idea what was going on… None at all. But that all changed in case 2 [when the camera moved closer].” (FG2*,* MS1)*.

All participants agreed that it could be important for both parties for the patient to see their physician. For the medical student, this aided in clinically assessing the patient and conducting a proper anamnesis. For the patient, it was believed to help establish a connection to the physician and to let them receive their diagnosis and care plan in a more compassionate manner.

While the new setting presented new challenges, all teams experienced a positive development between the first and second team training exercise. Two of the three teams expressed that they would find it easy to learn with a bit more practise, as articulated by a nursing student in the following excerpt:

*It feels like if you can do it [practise] a few times before doing it for real*,* you might be able to have a pretty good flow. (FG2*,* NS2)*

Interacting via telemedicine required new ways of communicating. In the excerpt below, a nursing student described how she thought she had made eye contact with the medical student who was participating remotely via the screen. She then realized that she had gone out of the camera angle and was not visible to the medical student.

*But it’s more difficult to make eye contact*,* or it was interesting to see what you were thinking*,* because we saw you out of the corner of our eyes*,* but then I realized that I was standing out of the sight for the camera. (FG3*,* NS1)*

In terms of SEIPS, the interaction between team members (the people) changed when one team member was not present in the room (the environment). The screen was described as a barrier, due both to difficulties in performing tasks and to the necessity of communicating directly with the screen (the tool). The work process could negatively affect the work outcome, as the physical absence of a physician from the room could hinder patient interactions. However, telemedicine was also described as a facilitator when it allowed for directed and safer communication.

## Discussion

In this study, we identified three themes: *challenging the dynamic of leadership*, *becoming familiar with a new setting*, and *finding new strategies to communicate*. The participants described aspects of interaction that became challenging when one of the team members was participating at a distance compared to an entirely physical face-to-face setting. One challenge was having the medical student only available via telemedicine; this meant that the nursing students had to perform not only their own tasks but also the examinations typically conducted by the medical student. Although the medical student provided them with instructions for performing these tasks, the nursing students lacked not only the knowledge but also the formal qualifications and practical skills to correctly carry them out. This underscores the necessity of education and integrating training for future healthcare professionals.

There is a significant demand for education using information communication technologies for healthcare students [22, 23]. However, a review by Chike-Harris et al. [22] found a lack of consistency in how telehealth was integrated into various healthcare educational curricula. While the content covered basic telehealth information, the depth and breadth of the content varied. The present study emphasizes the need for not only practical training in information communication technologies, but also preparing medical and nursing students for new tasks associated with working via telemedicine.

A second challenge and point for future consideration is how the medical students encountered issues with their position, as they found themselves taking a step back in their leadership roles. With the increasing use of telemedicine, alternative team structures have emerged in healthcare [24]. On the other hand, the nursing students formed a distinct team in the room with the patient and assumed a leadership role. Simulation-based team training can be aimed at enhancing teamwork by training interprofessional teams to improve skills such as leadership and communication [25, 26] and team training models are utilized in both undergraduate and postgraduate training. However, these models do not always consider the working conditions of rural distributed teams. Nevertheless, the medical and nursing students in this study confirmed that the team training sessions improved their performance in the second case compared to the first one.

Working via a screen also entails limited visibility, preventing team members from being seen before they enter or after they exit the screen. One of the nursing students initially thought she had made eye contact with the medical student who was participating remotely, but then realized that she had walked out of sight of the camera. If vision was optimized, this might assist leaders in maintaining a “hands off” approach. Working “hands off” has previously been shown to be associated with increased positive outcome and quality of teamwork in a study of trauma and resuscitation [22]. This was especially the case in less experienced teams, where direct leadership was found to be the more effective method, and the leader personally participating in tasks led to lower team performance [27].

Another point, also concerning vision, is that one team felt compelled to have face-to-face conversations despite the prevalence of using regular telephones for communication. Although this topic was not further explored during the interviews, one potential reason could be the importance of facial expressions in human communication [28]. Perhaps this group would have made a similar choice if given the option between a regular video conversation and a phone call, indicating that this is a matter of individual preference.

Telemedicine has seen widespread increase in usage since the start of the Covid-19 pandemic, and several studies have indicated that it leads to satisfied patients while also being cost effective [29, 30]. However, despite these benefits, telemedicine is not generally integrated into nursing [31] and medical education [32]. The participants in our interviews noted an improvement between the first and second scenario in the team training sessions, which is consistent with findings from a previous study conducted in the USA [33]. Hindman et al. [33] demonstrated an enhanced practical examination performance in telemedicine by medical students with telemedical training compared to a control group with a standard curriculum. Moreover, medical students with telemedical training reached the same diagnoses as the control group while utilizing fewer tests.

An editorial published in the Lancet in 2020 [34] noted that over the previous decade the focus had been on transitioning patient care from hospitals to primary health care centres. The focus is now expected to shift towards providing care in patients’ homes, utilizing tools such as mobile stroke units and mobile applications. If this trend holds true, then having increased knowledge and practise-based experience of telemedicine may prove important for future medical and nursing students. The global adoption of information communication technology in clinical practise also means that health care educators will need to integrate innovative ways to introduce and engage students into telehealth.

Using the theoretical framework of the SEIPS model as a lens, when describing the results of this study we were able to discuss interactions between nursing and medical students (people) via telemedicine (tool and environment) during team training (task) in relation to the work process and work outcomes. This theoretical framework helps clarify how the interaction between the nursing and medical students through telemedicine could both positively and negatively affect the work process and work outcomes.

### Strengths and limitations

In this study, we utilized focus group interviews to capture the team members’ descriptions and narratives of the team training sessions [35]. A notable strength of focus group interviews lies in their demonstrated ability to facilitate discussions on sensitive topics. To confirm the t*rustworthiness* of the research process, continuous discussions were conducted among the authors, each possessing diverse methodological expertise. This approach aligns with Creswell’s emphasis on the importance of incorporating multiple perspectives to ensure *credibility* and rigor in qualitative research [36]. To increase the *credibility* and *authenticity* of the analysis, excerpts from the interviews have been inserted into the text to verify the *accuracy* of the findings. One limitation of this study is that the sample size was small and all participants were from the same university. However, the findings from this study indicate that the team training sessions were experienced as valuable by the students, and suggest that further research is required to determine if more simulations should be offered. During the focus group interview, the interviewers ensured that the participants could speak, and that time was equitably distributed among the interviewees.

## Conclusions

The participants in this study found that teamwork via telemedicine had both advantages and challenges. Roles within the team shifted; for the medical student, the overview of the situation was improved and contributed to a reduced workload, while the nursing students faced increased responsibility and workload. The present findings suggest the use of simulation-based team training to increase knowledge of practicing teamwork via telemedicine in medical and nursing education.

## Electronic supplementary material

Below is the link to the electronic supplementary material.

Supplementary Material 1

## Data Availability

No datasets were generated or analysed during the current study.

## Abbreviations

ABCDE: Airway, Breathing, Circulation, Disibility and Exposure
CPR: Cardiac and Pulmonary Resuscitation
ECG: ElectroCardioGram
ER: Emergency Room
FG: Focus Group
MS: Medical Student
NS: Nursing Student
SPEIS: The Systems Engineering Initiative for Patient Safety model
X-ray: Electromagnetic radiation
