# Supplementary material for: Interprofessional team training via telemedicine in medical and nursing education
Source: BMC Med Educ. 2024 Oct 8;24:1110. doi: 10.1186/s12909-024-06104-8 (PMC11463107; doi:10.1186/s12909-024-06104-8)
Supplement: Supplementary file 1 — Supplementary Material 1 [file 12909_2024_6104_MOESM1_ESM.docx]

**Interview guide**

If you think about today's team training:

“What was your role in the team?“

“How did your role evolve when the team was not in the same place?”

“How did you perceive leadership in the distributed setting?”

Was there difficulties?

follow-up questions such as “Could you please elaborate on this?”

“How did you perceive the collaboration in the distributed setting?”

Was there difficulties?

follow-up questions such as “Could you please elaborate on this?”

“Can you tell me how you experienced communication in the distributed setting?”

Was there difficulties?

follow-up questions such as “Could you please elaborate on this?”
